# Importance of clinical parameters for cultivation of critical care thinking by online teaching of critical care medicine

**DOI:** 10.1186/s12909-023-04435-6

**Published:** 2023-06-30

**Authors:** Pan Pan, Min Zheng, Hongbo Luo, Jinbang Liu, Lina Li, Longxiang Su

**Affiliations:** 1 College of Pulmonary and Critical Care Medicine, Eighth Medical Center, Chinese People’s Liberation Army General Hospital, Beijing, 100089 China; 2 Department of Critical Care Medicine, State Key Laboratory of Complex Severe and Rare Diseases, Peking Union Medical College Hospital, Chinese Academy of Medical Science and Peking Union Medical College, Beijing, 100730 China

**Keywords:** Critical care medicine, Parameter, Teaching, Clinical thinking

## Abstract

**Background:**

The teaching of critical care medicine is a very important task, especially during the COVID-19 pandemic. The understanding of critical care parameters is the foundation and core, which is conducive to the formation of clinical thinking. This study is to evaluate the training effect of teaching of critical care parameters based on an online platform, and explore the teaching methods of critical care medicine that can help to cultivate trainees’ clinical thinking and practical ability.

**Methods:**

Questionnaires were released before and after the training through the official new media platform “Yisheng” application (APP) of China Medical Tribune involving 1109 participants. The trainees who filled in the questionnaire in APP and received training were randomly selected as the investigated population. Statistical description and analysis were carried out using SPSS 20.0 and Excel 2020.

**Results:**

The trainees were mainly attending physicians in tertiary hospitals and above. Among all critical care parameters, trainees paid more attention to critical hemodynamics, respiratory mechanics, severity of illness scoring systems, critical ultrasound, and critical hemofiltration. The degree of satisfaction with the courses was high, especially the course of critical hemodynamics was scored the highest. The trainees believed that the course contents were of great help to clinical work. However, no significant difference was found in the trainees’ understanding or cognition of the connotation of the parameters before and after the training.

**Conclusion:**

Teaching of critical care parameters based on an online platform is conducive to improving and consolidating the clinical care ability of trainees. However, it is still necessary to strengthen the cultivation of clinical thinking in critical care. In the future, the integration of theory with practice must be strengthened in clinical practice, ultimately achieving the homogeneous diagnosis and treatment of patients with critical illness.

**Supplementary Information:**

The online version contains supplementary material available at 10.1186/s12909-023-04435-6.

## Background

Critical care medicine in the Chinese mainland has developed for only 40 years. It is a young and also very important discipline. Due to the late start of critical care medicine and uneven distribution of medical resources in China, most physicians engaged in critical care medicine came from non-critical care medicine majors, and did not receive specialized training in critical care medicine, leading to uneven levels of critical treatment in different regions of China [1]. Therefore, it is extremely urgent to establish and improve the discipline system of critical care medicine in China and carry out standardized and homogeneous training for critical care majors. Online teaching has grown tremendously during the COVID-19 period. For China's curtical care medicine, this is nothing more than a very good opportunity. But how to use this teaching form, or the effect of this teaching form needs to be considered.

A parameter means a quantity whose value is selected for the particular circumstances and in relation to which other variable quantities may be expressed. In clinical work, there are a large number of parameters to reflect the basic characteristics and status of patients, including vital signs, clinical laboratory examinations, imaging data, and so on. For the critically ill patients, there are more types of parameters [2]. Moreover, the relevance of the parameters is stronger, and continuous and dynamic changes are more intense. Mastering these parameters is the key to accurately judging the patients’ condition and make correct treatment decisions. Whether to accurately use these parameters in clinical work requires certain clinical thinking to integrate these parameters [3]. In this study, basic training of critical care parameters was carried out based on the clinical needs of Chinese critical care physicians, and relevant questionnaires were designed before and after the training, aiming to explore the specific needs of critical care physicians for training content, clarify the practice effect after training, and seek the bottleneck that restricts the development of Chinese critical care medicine from training to clinical practice.

## Methods

### Course design and background

To ensure the master of critical care staff of the basic knowledge of critical care medicine, improve their clinical work, and strengthen the homogeneous management of critically ill patients, a small video class of critical care medicine was set up on the official new media platform “Yisheng” of China Medical Tribune. For better addressing the training needs of front-line clinical workers, a questionnaire investigation was conducted on the course content about critical care parameters, and the course was optimized accordingly. The course was launched on August 23, 2022 in the form of a training camp (one class a day, about 30–40 min per class), with a total of 30 classes, lasting for one month.

### Investigated population

The questionnaires were released through the official new media platform “Yisheng” application (APP) of China Medical Tribune. Before the course, the investigation was performed from April 22 to May 4, 2022. After the course, the investigation was performed from September 23 to September 30. The trainees who filled in the questionnaire in APP and received training were randomly selected as the investigated population.

### Questionnaire implementation

The investigation before the course involved the basic information of trainees, cognition of critical parameters, and training needs for critical parameters. The investigation after the course included the basic information of trainees, cognitive changes after training, training experience, and course evaluation.

### Statistical analysis

This study is a descriptive observational study. Results for continuous variables with normal distributions are given as means ± standard deviations (SD). Student's t-test was used to compare means between two groups. Analysis of variance (ANOVA) was used to compare means among multiple groups. Results for qualitative variables were expressed as percentages and compared between groups using a Chi-square test. All parameters were statistically described and analyzed using SPSS 20.0 and Excel 2020.

## Results

### Participants

This study was divided into two parts: Pre-training and post-training investigation. A total of 664 individuals participated in the pre-training investigation, of which 60.17% were from tertiary hospitals and 44.83% were attending physicians. A total of 445 individuals were surveyed after the course, of whom 67.29% were from tertiary hospitals and 48.14% were attending physicians. From the occupational analysis of the personnel participating in the training, the number of trainees in critical care medicine was the largest. About 48.13% of the respondents completed 20–30 training classes in the training camp. There were no statistical differences between the population participating in the investigation before and after the training.

### Pre-training investigation

**Fig. 1:**
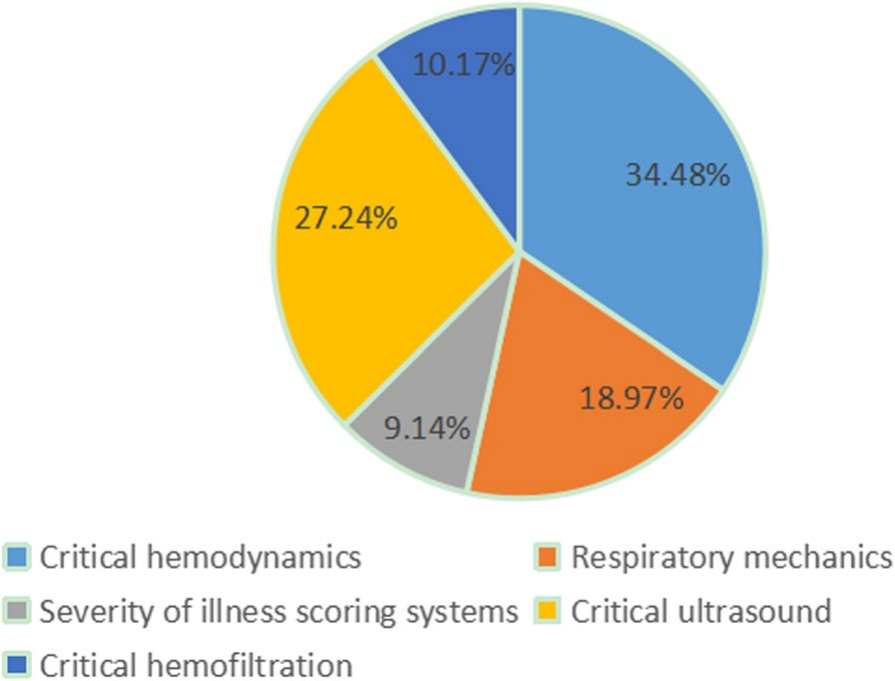
Distribution of trainees’ interests in the preliminary proposed directions of the course

**Fig. 2:**
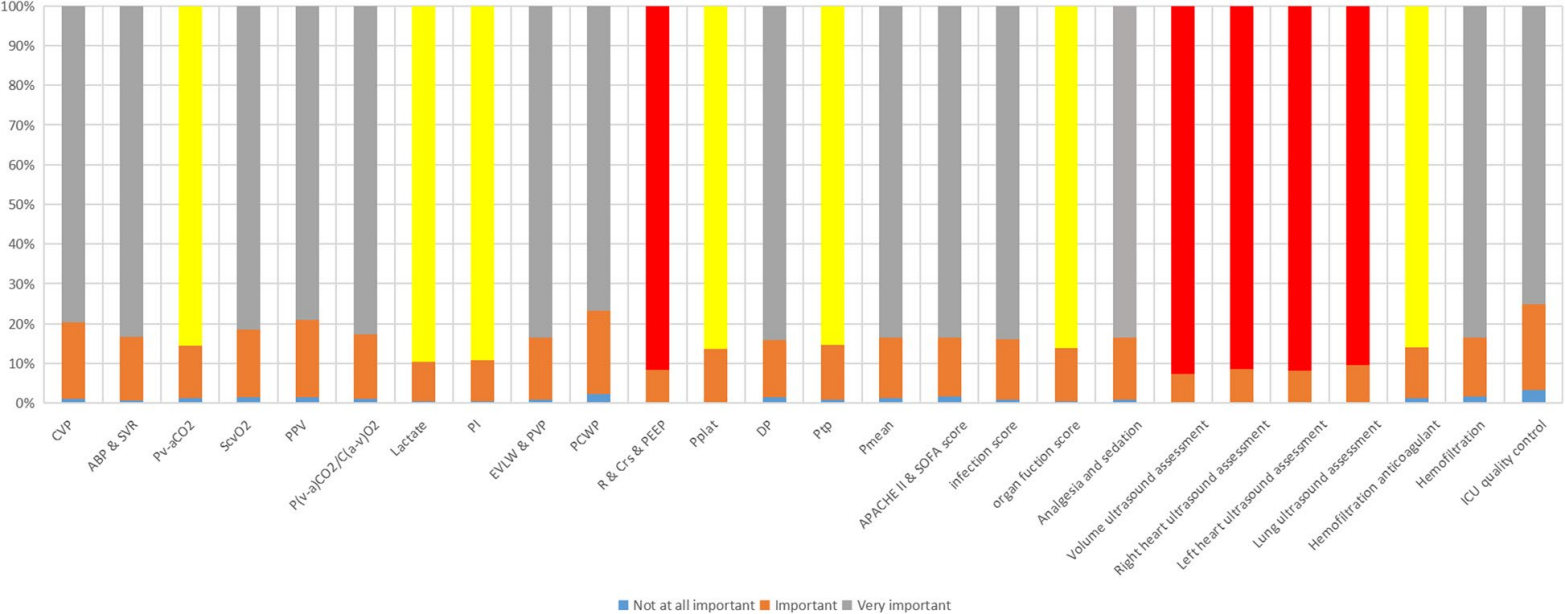
Scores of the specific contents of the preliminary proposed directions of the course. Red color represents supported by more than 90% of the trainees, and yellow represents supported by more than 80% of the trainees

Before the training, the core critical Cooperative for American Relief Everywhere parameters were investigated to screen the trainees’ attention to the course. Critical hemodynamics, respiratory mechanics, severity of illness scoring systems, critical ultrasound, and critical hemofiltration were preliminarily proposed, and the attention to the contents was evaluated (Fig. 1). In these five directions, the detailed contents were further set up for trainees to score (Fig. 2), the final training program (Supplemental Table) was determined, and the courses were recorded. The training video was launched on August 23, 2022.

### Post-training evaluation

**Fig. 3:**
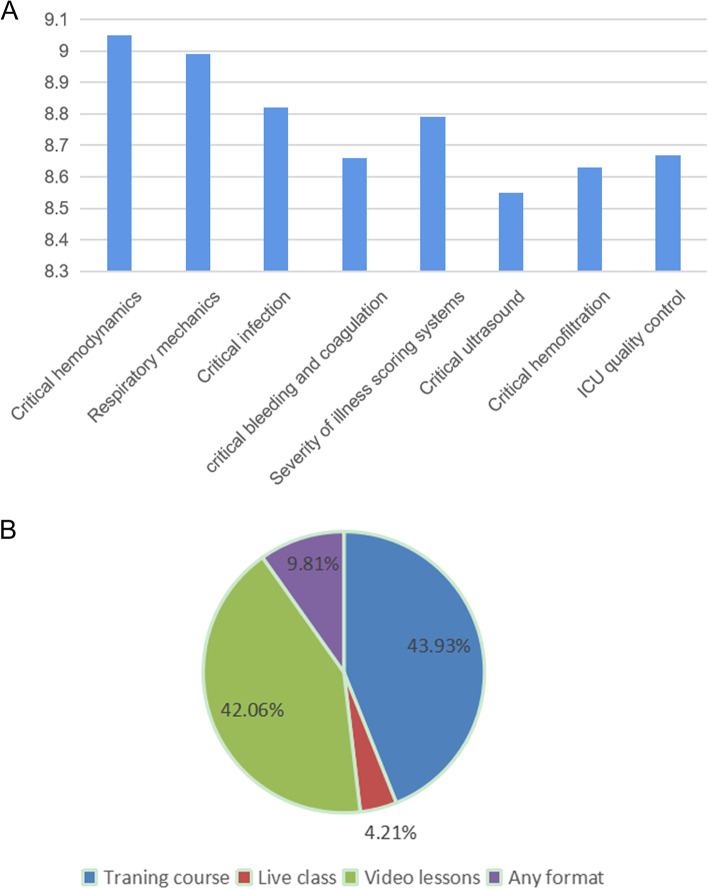
Course evaluation. **A** Overall score of the course; **B** Support for online teaching

After the completion of the course, the satisfaction and course form were investigated. The course contents were scored according to the training directions (Fig. 3A), and the course form was investigated (Fig. 3B). From the data analysis, critical hemodynamics was the most important learning direction. The training camp is everyone’s favorite learning form.

### Cognitive changes before and after training and training effect

**Fig. 4:**
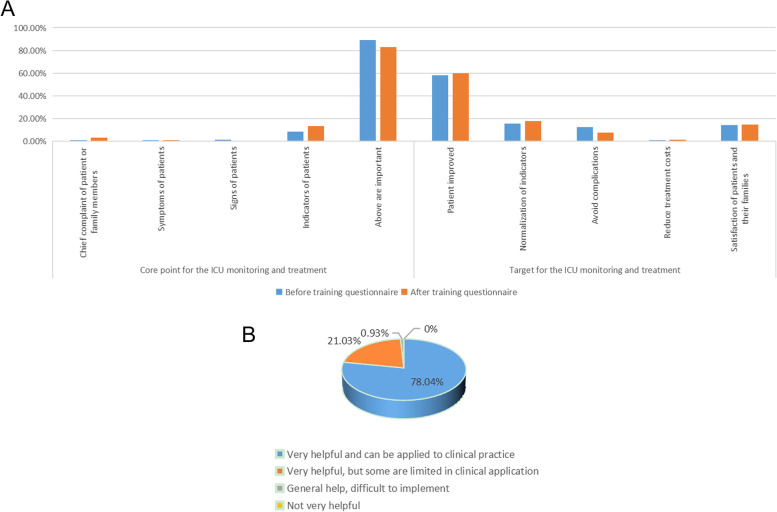
Cognitive changes of trainees before and after training and training effect evaluation. **A** Before and after training; **B** Training effect evaluation

Whether the trainees had a deeper understanding of parameters and whether it was helpful for their clinical work were clarified. Three questions were designed in the pre-training questionnaire, two of which were re-tested in the post-training questionnaire. It was found that the questionnaire respondents were very clear about the goal of critical treatment, including judging the acute reversible critical state, identifying some potential high-risk critically ill patients (such as major surgery with many basic diseases), determining the acute exacerbation stage of some chronic diseases, and mastering the treatment of end-stage diseases except for advanced cancers or chronic diseases (90.69%). The changes in the cognition of the core of critical care monitoring and treatment and the definition of critical treatment goals before and after the course are shown in Fig. 4A, without statistical differences before and after the training about the cognition. However, 78.04% of trainees thought that it was helpful and can be applied to the clinical practice. Figure 4B displays the clinical training effect of this course.

## Discussion

This study focused on the teaching of the parameters for critical care treatment. Before and after the teaching, the courses were designed and tracked through questionnaires. According to the investigation results, the grassroots critical care physicians had a strong demand for basic knowledge, could put forward needs, and were influenced and helped in clinical behaviors through the training. However, the cognition of the participants on critical care thinking still needs to be improved.

Intensive care units (ICU) in China are developing rapidly. According to the three national ICU censuses in 2006, 2011, and 2015, the number of hospitals equipped with the Department of Critical Care Medicine in China has increased from more than 1000 to nearly 4000, the number of ICU physicians nationwide has increased to 63,605, and the number of ICU nurses has increased to more than 100000 [4]. The great challenge faced by the increasing number of ICU practitioners is how to form standardized cognition and workflow [5]. Our investigation showed that ICU practitioners had a great demand for basic knowledge training, and we also observed the weak foundation of the practitioners during the training, especially in critical hemodynamics, critical ultrasound, and respiratory mechanics, for which the trainees presented high enthusiasm. In these parts, the respondents were interested in the measurement of respiratory mechanics and the basic operation of critical ultrasound. Therefore, the demonstration of traditional respiratory mechanic measurement was added to the course of respiratory mechanics, and the ACCUE process was supplemented to the ultrasound course for the use of front-line clinical personnel [6]. Of course, according to the characteristics of different training programs, we should carry out relevant offline training auxiliary programs in conjunction with online teaching to deepen the understanding and application of indicators. For example, the ultrasound course is a highly practical specialty, and we should conduct hand-on teaching and simulation teaching if conditions permit.

The feedback after the training indicated that all course scores were more than 8.5, especially the hemodynamic score could reach more than 9.0, suggesting that everyone recognizes the course (the full score of course evaluation is set to 10 points). Online teaching has become the mainstream training mode in the post-coronavirus disease 2019 (COVID-19) era, which provides a new idea for the training and teaching of critical care medicine [7, 8]. Our teaching attempt has greatly affirmed this teaching mode. This is also a very important form of learning in the background of the COVID-19 epidemic in China [9]. It is generally acknowledged that training camps and recording and broadcasting courses can facilitate to well and systematically learn and review relevant knowledge, and are widely loved by trainees. Moreover, Yisheng platform has also been recognized as an excellent official course promotion platform in the Chinese mainland. As for the training effect, 78.04% of the respondents believed that it was helpful to clinical practice and could be directly applied in clinical practice. However, it is a pity that this investigation did not reveal difference in the cognition of critical care parameters before and after the training, which may result from some inherent misconceptions about parameters in the past,. Therefore, updating critical thinking requires some time to precipitate and practice.

The core of critical monitoring and treatment is the parameters of critically ill patients, and the goal of critical care treatment is the normalization of parameters, which is an important basis for our training. Parameters measured clinically or obtained through the laboratory are all derived from the clinical practice, and are a part of the clinical manifestations and the extension of clinical observation. As a specific clinical language, parameters have their own unique status and significance. Each parameter has its own connotation. Different parameters can complement each other, but cannot replace each other. As long as the measurement is accurate, each parameter represents the objective existence and has applicable value. Only a clear understanding of each parameter can make clear what the goal of treatment is and make more accurate progress towards the treatment goal [10]. The relationship between goals and objectives in critical care medicine is the core thinking of critical care medicine. Only by mastering the essence of critical care medicine thinking, can we effortlessly deal with the problems in treatment.

There were several limitations to this study. First, the crowds of the investigation of this study may not the same group of trainees before and after the questionnaire survery. Although there were no statistical differences in the crowds before and after the investigation from job titles, majors, hospital grades, ect., there may be an impact on the results. Second, this study is a survey of basic training. The content of the training has certain limitations. Professional intensivist should provide more professional and deeper training and understand their needs and functions. It is important to carefully consider its impact on the development of clinical skills and to find ways to supplement online teaching with hands-on training and real-world experience. That is to say, online teaching has its advantages, it is more convenient than traditional teaching, and students can learn repeatedly. But it cannot replace the practical nature of clinical medicine itself. Therefore, while studying, we must pay attention to the practice of teaching, otherwise it is not easy to form mature clinical thinking. Simulation teaching and case teaching are very good forms. How to integrate them into the current online new media teaching form is worth exploring in the future.

## Conclusion

The teaching of critical care medicine in China needs to start from the foundation, and the training of critical care parameters is a good start. Moreover, online teaching provides a convenient and new way for post-COVID-19 training, and is worthy of extensive promotion. During the training, we need to pay attention to the design of courses, and run the training of critical thinking through the whole process of critical care training. Of course, training is only the teaching of theoretical knowledge. We must strengthen the integration of theory with practice in clinical practice, and emphasize the directivity and importance of parameters. Through the training of the precise application of parameters, precise treatment can be performed for patients, ultimately achieving the homogeneous diagnosis and treatment for critically ill patients.

## Supplementary Information

**Additional file 1:** **Table S1. **The training program including 30 topics.

## Data Availability

The datasets used and/or analysed during the current study available from the corresponding author on reasonable request.
