# Supplementary material for: Importance of clinical parameters for cultivation of critical care thinking by online teaching of critical care medicine
Source: BMC Med Educ. 2023 Jun 30;23:485. doi: 10.1186/s12909-023-04435-6 (PMC10311842; doi:10.1186/s12909-023-04435-6)
Supplement: Supplementary file 1 — Additional file 1: Table S1. The training program including 30 topics. [file 12909_2023_4435_MOESM1_ESM.docx]

**Table 1 The training program including 30 topics**

| **Classification** | **Course contents** |
| --- | --- |
| **Critical hemodynamics** | Central venous pressure |
|  | Artieral blood pressure & systemic vascular resistance |
|  | Venous to arterial carbon dioxide partial pressure difference |
|  | Central venous oxygen saturation |
|  | Pulse pressure variation |
|  | Venous-to-arterial carbon dioxide difference to arteriovenous oxygen content difference ratio |
|  | Lactate |
|  | Perfusion index |
|  | Extravascular lung water & Pulmonary vascular permeability |
|  | Pulmonary capillary wedge pressure |
| **Respiratory mechanics** | Respiratory resistance and compliance |
|  | Positive end-expiratory pressure |
|  | Plateau pressure |
|  | Driving pressure |
|  | Transpulmonary pressure |
|  | Mean airway pressure |
| **Critical infection** | Severe infection biomarkers |
|  | Pathogenic microorganism and drug sensitivity test |
| **Critical bleeding and coagulation** | Critical bleeding and coagulation parameters |
| **Severity of illness scoring systems** | Acute Physiology and Chronic Health Evaluation II & Sequential Organ Failure Assessment score |
|  | Infection score |
|  | Organ fuction score |
|  | Analgesia and sedation score |
| **Critical ultrasound** | Volume ultrasound assessment |
|  | Heart ultrasound assessment |
|  | Lung ultrasound assessment |
|  | ACCUE protocol |
| **Critical hemofiltration** | Hemofiltration mechenism |
|  | Hemofiltration anticoagulant |
| **ICU quality control** | ICU quality control |

ICU: Intensive care unit.
